# Can an individual with low frequency hearing in the candidate ear benefit from a cochlear implant even if they have normal hearing in the other ear?

**DOI:** 10.1007/s00405-023-08011-y

**Published:** 2023-05-24

**Authors:** Artur Lorens, Piotr Henryk Skarzynski, Anita Obrycka, Henryk Skarzynski

**Affiliations:** 1 World Hearing Center, Institute of Physiology and Pathology of Hearing, Kajetany/Warsaw, Poland; 2 Heart Failure and Cardiac Rehabilitation Department, Medical University of Warsaw, Warsaw, Poland; 3 Institute of Sensory Organs, Kajetany, Poland

**Keywords:** Cochlear implant, Partial deafness, Single-sided deafness, Hearing preservation, Quality of life

## Abstract

**Purpose:**

To determine hearing preservation and subjective benefit after cochlear implant (CI) surgery in patients with low frequency hearing in the ear to be implanted (i.e., they have partial deafness, PD) and close to normal hearing in the other.

**Methods:**

There were two study groups. The test group was made up of 12 adult patients (mean age 43.4 years; SD 13.6) with normal hearing or mild hearing loss in one ear, and with PD in the ear to be implanted. The reference group consisted of 12 adult patients (mean age 44.5 years; SD 14.1) who had PD in both ears and who underwent unilateral implantation in their worse ear. Hearing preservation was assessed 1 and 14 months after CI surgery using the Skarzynski Hearing Preservation Classification System. The APHAB questionnaire was used to evaluate the benefit from the CI.

**Results:**

The differences in HP% between the groups were not significant: mean hearing preservation (HP%) in the test group was 82% one month after CI surgery and 75% some 14 months after implantation; corresponding results in the reference group were 71% and 69%. However, on the APHAB background noise subscale, the benefit in the test group was significantly larger than in the reference group.

**Conclusion:**

To a large extent it was possible to preserve low-frequency hearing in the implanted ear. This means that individuals with low frequency hearing in the implanted ear (partial deafness) and with normal hearing in the other generally received more benefits from cochlear implantation than did patients with partial deafness in both ears. We conclude that residual low frequency hearing in the ear to be implanted should not be considered a contraindication for a CI in a patient with single-sided deafness.

## Introduction

With advances in surgical technique and electrode design, preserving residual hearing has now become possible [1], and this has expanded the candidacy criteria to include patients with lesser degrees of hearing loss [2]. Skarżyński et al. [1] reported successful hearing preservation in a patient with partial deafness (PD), meaning that thresholds in the low frequency (LF) region were normal or slightly elevated, although there was almost total deafness at higher frequencies. This case was probably the first reported of implantation in an ear with normal hearing in the LF range. Since then, HP in PD cases following CI surgery has been confirmed in many studies [3–16].

Unfortunately, direct comparison of many HP case series is difficult in that for many years there was no standardized protocol for inclusion criteria for those with residual hearing and no standard method for reporting HP outcomes. In the literature, different definitions have been used for defining HP [10, 17, 18].

To overcome the lack of an accepted HP standard, Skarzynski et al. [19] proposed a comprehensive HP classification system that does not depend on the user’s preoperative hearing levels and is therefore suitable for reporting the HP of all patient groups. The system is based on the formula: HP% = [1 – (PTA postoperative – PTA preoperative) / (PTA max – PTA preoperative)], where PTA max is the average maximum output of the reference audiometer. There are also four categories of HP% proposed: category HP1 is complete or near-complete preservation with an HP of more than 75%; HP2 is partial preservation with an HP of 25–75%; HP3 is minimal HP with an HP of 0–25%; and HP4 is loss of hearing/no hearing in which no measurable hearing is detected after implantation.

The Skarzynski HP classification system makes it possible to more easily compare study outcomes and perform meta-analysis of the data. In most HP studies, at least the low frequencies (0.25, 0.5, and 1 kHz) are reported, so that Snels et al. [3] was able to use the Skarzynski et al. [19] HP classification system for a meta-analysis. Snels calculated HP% values from the results of 26 case series, and the mean HP% for the combination of a round window approach and a straight electrode was estimated to be 82.2% one month postoperatively and 69.0% 12 months postoperatively. The authors concluded that HP in CI surgery is feasible in patients with partial deafness and that extending qualification criteria is justifiable [3].

The current study describes our experience of CI with HP in a unique group of patients: they had low-frequency (LF) hearing in the ear to be implanted (they had PD) and normal or close to normal hearing (NH) in the other. Despite having LF hearing in the ear to be implanted, these patients still met the generally acceptable audiometric criteria for single sided deafness (SSD) cochlear implantation, as their pure tone average (PTA) threshold in the ear to be implanted was 90 dB or greater while the PTA in the NH ear was no worse than 30 dB. We therefore called them the SSD-PD group.

The study aimed to determine whether HP would be successful in this unique population (the test group), and, if so, what the potential benefit of the CI might be. HP and CI benefit were also evaluated in a reference group: patients with PD in both ears who also underwent unilateral cochlear implantation at about the same time as the study group. The motivation of the study was to gauge what improvement a CI gave to patients with low frequency hearing in the candidate ear when they had normal hearing in the other ear.

## Material and methods

**Fig. 1:**
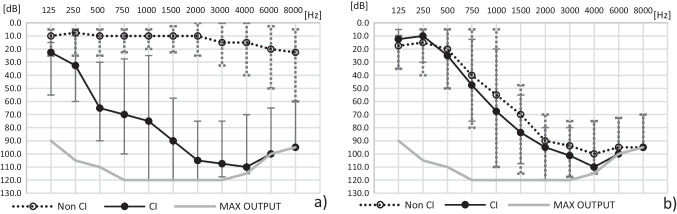
Median preoperative threshold for the SSD-PD group (**a**) and for the PD group (**b**). Whiskers indicate first and third quartiles

A group of 12 adult patients with normal hearing or mild hearing loss in one ear, and with partial deafness (hearing thresholds at 0.5 kHz better than 80 dB) in the contralateral ear, were included in the study (SSD-PD or test group). All patients fulfilled the criteria for single-sided deafness (SSD) [20]. Median audiometric preoperative thresholds of the SSD-PD group are shown in Fig. 1a. The mean aided monosyllabic speech discrimination for the ear to be implanted, tested at 65 dB SPL presentation level was 27.3% (SD 25.6).

Patients were implanted at the Institute of Physiology and Pathology of Hearing, Poland, in their poorer-hearing ear (the ear with partial deafness) using the six-step round-window approach surgery developed by Skarzynski et al.[17]. The six-step procedure minimizes trauma to the delicate cochlear structures that remain [17]. In order to avoid loss of low-frequency hearing, short electrodes were used in all patients, except one.

The age at CI surgery ranged from 18 to 61 years (mean age 43.4; SD 13.6). All patients fulfilled 14 months of CI follow-up. Following surgery, the speech processor was fitted for each subject according to the CI manufacturer’s guidelines. Demographic data of the SSD-PD group is presented in Table 1.

**Table 1:** Subject data

	Number of subjects	
	SSD-PD	PD
Gender		
Male	6	5
Female	6	7
Etiology		
SIHL	2	0
Unknown	6	7
Otosclerosis	1	1
Meningitis	0	1
Noise exposure	0	1
Head trauma	2	0
Virus infection	1	0
Middle ear infection	0	2
Hearing loss type		
Progressive	1	11
Sudden	11	1
Tinnitus before CI		
Yes	11	5
No	1	7
CI ear		
Right	5	5
Left	7	7
CI type		
Concerto	4	2
Sonata	7	10
Synchrony	1	0
Electrode type		
Flex 20	3	3
Flex 24	7	9
Flex 28	1	0
Medium	1	0
Processor type		
Opus2	1	0
Sonnet	9	11
Rondo	2	1

*SIHL* sensorineural idiopathic hearing loss, *CI*  cochlear implant

The second (reference) group consisted of 12 adult patients with partial deafness in both ears (PD group), who underwent unilateral implantation at the Institute of Physiology and Pathology of Hearing using the same surgical procedure (the six-step round-window approach). The patients were matched according to age at implantation (mean age 44.5; SD 14.1; range 20–64), hearing loss in the ear to be implanted (which had largely retained low frequency hearing: hearing thresholds for 0.5 kHz of up to 80 dB), gender, and side of implantation. In addition, their dates of implantation were in the same range as the SSD-PD group. Demographic data of the PD group is also presented in Table 1, and their median audiometric preoperative thresholds are shown in Fig. 1b. The mean aided monosyllabic speech discrimination for the ear to be implanted, tested at 65 dB SPL presentation level was 44.6% (SD 18.6).

Hearing preservation (HP) was assessed using the Skarzynski hearing preservation formula and classification [19]. The degree of preservation, HP%, was evaluated in both groups twice, 1 month and 14 months after surgery. The data were also analyzed in terms of the four HP categories.

The APHAB (Abbreviated Profile of Hearing Aid Benefit) questionnaire was used to evaluate the benefit from cochlear implantation [21]. All four subscales were analysed: ‘ease of communication’ (EC), which assesses speech understanding under relatively favourable conditions; ‘background noise’ (BN), which relates to communication in noisy settings; ‘reverberation’ (RV), associated with communication in reverberant places; and ‘aversiveness’ (AV), which evaluates the unpleasantness of environmental sounds. The first three of these subscales provide a ‘global score’ (GS), which is the mean of the EC, RV, and BN scores. The APHAB was administered 14 months after the CI surgery, and the APHAB results from the test group (SSD-PD) were compared with the results from the reference group (PD).

The hypothesis of a normal distribution of the data was evaluated using a Shapiro–Wilks test. Two-way repeated measures ANOVA was used to determine (1) the effect of time on hearing preservation, HP%, and (2) whether HP% differed significantly between the SSD-PD and PD groups. Two-way repeated measures ANOVA was also used to test (1) the effect of intervention (cochlear implantation) on APHAB results, and (2) whether the APHAB results differed significantly between study groups. The level of significance was set at α = 0.05.

The study was designed and conducted according to the Declaration of Helsinki and the study protocol was reviewed and approved by the Institutional Review Board.

## Results

**Fig. 2:**
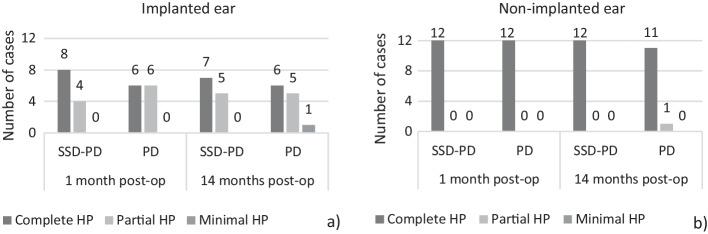
Histograms of hearing preservation categories, 1 and 14 months after CI surgery, in 12 SSD-PD cases and 12 PD cases. **a** Implanted ear; **b** Non-implanted ear

Figure 2a shows that, 1 month after CI surgery, there was complete or partial hearing preservation in all implanted ears, both in the test group (SSD-PD) and in the reference group (PD). However, some 14 months postoperatively, there was a deterioration from ‘complete HP’ to ‘partial HP’ in one case from the SSD-PD group; similarly, in one case from the PD group there was a deterioration from ‘partial HP’ to ‘minimal HP’. As expected, in the non-implanted ear, hearing preservation was classified as complete in all cases 1 month postoperatively and in all cases (except one) from the PD group 14 months after surgery.

Mean hearing preservation HP% for the implanted ear in the SSD-PD group was 82% one month after CI and 75% some 14 months after implantation; corresponding results in the PD group were 71% and 69%. Two-way repeated measures ANOVA showed no significant effect of time (*F*(1,22) = 1.46; *p* = 0.24); no group effect (*F*(1,22) = 0.82; *p* = 0.38); and no interaction between those variables (*F*(1,22) = 0.4; *p* = 0.53). This indicates that hearing preservation in the SSD-PD and PD groups did not differ, and that hearing thresholds were stable over 14 months of observation in both groups.

**Fig. 3:**
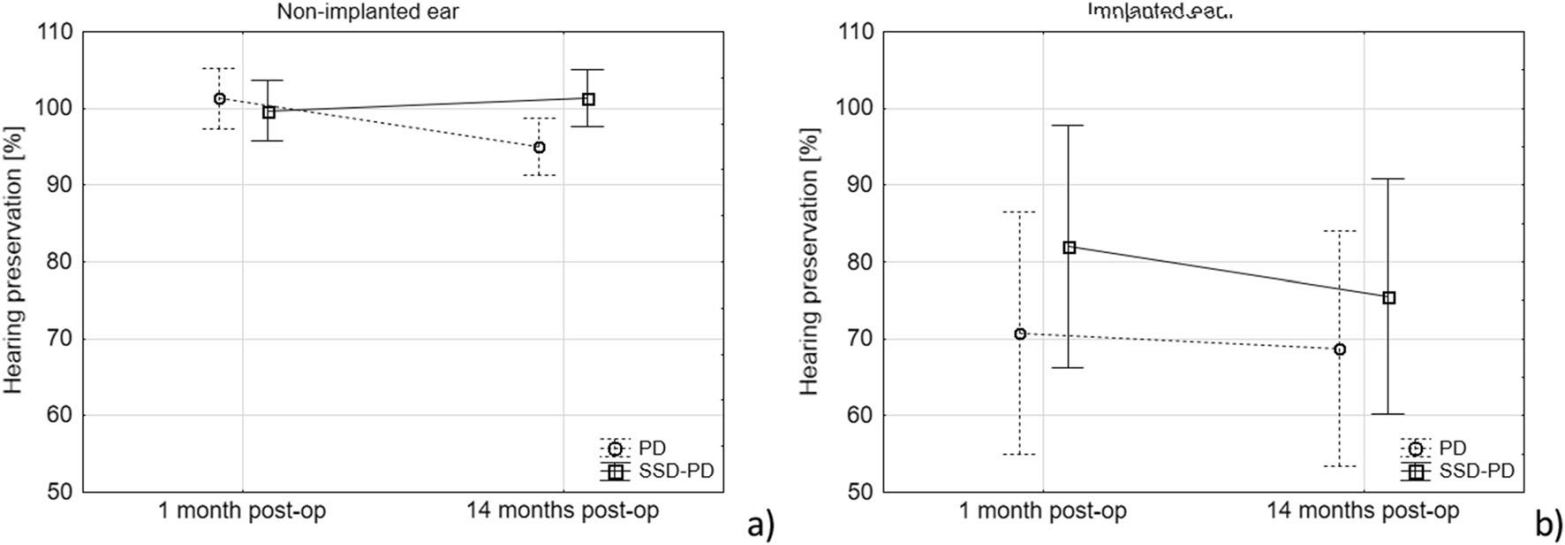
Hearing stability in the non-implanted ear (**a**), and hearing preservation in the implanted ear (**b**), calculated using the HP formula

We also analysed the stability of hearing thresholds in the non-implanted ear using the same formula and classification system. Analysis showed no significant effect of time (*F*(1,22) = 1.46; *p* = 0.24); no group effect (*F*(1,22) = 0.82; *p* = 0.38); and no interaction between variables (*F*(1,22) = 0.4; *p* = 0.53). The results are plotted in Fig. 3.

**Fig. 4:**
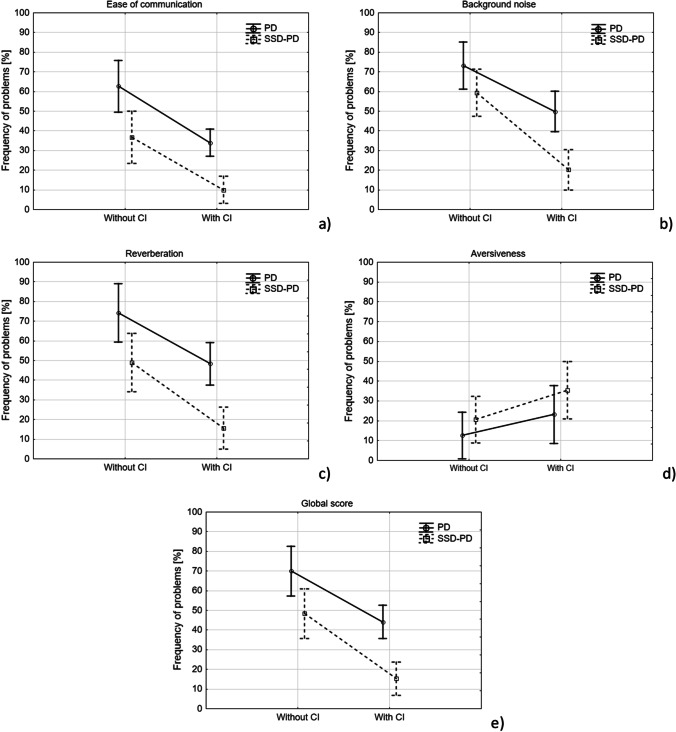
Results of APHAB questionnaire for: **a** Ease of communication sub-scale, **b** Background noise sub-scale, **c** Reverberation sub-scale, **d** Aversiveness sub-scale, and **e** Global score. Points indicate mean frequency of problems for the tested subgroups and conditions; whiskers show 95% confidence intervals

The hearing benefit from cochlear implantation was calculated as the difference between ‘Without CI’ and ‘With CI’ conditions for each APHAB subscale and for the global score. The mean benefit in the SSD-PD group was: for EC = 26.7 p.p. (SD = 18.7), for BN = 39.1 p.p. (SD = 18.6), for RV = 33.4 p.p. (SD = 24.9), for AV =  − 14.9 p.p. (SD = 22.9), and for GS = 33.1 p.p. (SD = 20.1). The mean benefit in the PD group was: EC = 28.7 p.p. (SD = 20.4), BN = 23.3 p.p. (SD = 17.2), RV = 25.9 p.p. (SD = 21.9), AV =  − 10.5 p.p. (SD = 11.1), and GW = 25.9 p.p. (SD = 18.3). Detailed results for both groups are presented in Fig. 4.

Moreover for the EC sub-scale, two-way repeated measures ANOVA revealed a significant main effect of patient group (*F*(1,22) = 17.4; *p* < 0.001) and a significant main effect of CI use (*F*(1,22) = 48.2; *p* < 0.001). There was no statistically significant interaction between the effects of patient group and CI use (*F*(1,22) = 0.065; *p* = 0.8). Similar results were obtained for the RV subscale and for GS: a significant main effect of patient group (for RV: *F*(1,22) = 15.2; *p* < 0.001; for GS: *F*(1,22) = 16.8; *p* < 0.001) and a significant main effect of CI use (for RV: *F*(1,22) = 38.5; *p* < 0.001; for GS: *F*(1,22) = 56.6; *p* < 0.001). There was no statistically significant interaction between the effects of patient group and CI use (for RV: *F*(1,22) = 0.61; *p* = 0.44; for GS: *F*(1,22) = 0.84; *p* = 0.37). This indicates that for the EC and RV sub-scales, as well as for GS, a significant benefit from CI use was observed regardless of the patient group; however, the reported frequency of problems was higher in the PD group compared to the SSD-PD group in both conditions: with CI and without CI (Figs. 4a, c, e).

For the BN sub-scale, ANOVA revealed a significant main effect of patient group (*F*(1,22) = 10.6; *p* = 0.003), a significant main effect of CI use (*F*(1,22) = 72.9; *p* < 0.001), and a significant interaction between the effects of patient group and CI use (*F*(1,22) = 4.69; *p* = 0.04). For the BN sub-scale, the frequency of problems without a CI was similar for both the PD and SSD-PD groups. At the same time, with a CI, the frequency of problems significantly decreased in both groups, and was significantly lower in SSD-PD patients compared to PD patients (Fig. 4b).

For the AV sub-scale, we only found a significant main effect of CI use (*F*(1,22) = 11.9; *p* = 0.002). There was no effect of patient group (*F*(1,22) = 1.5; *p* = 0.23) or interaction between patient group and CI use (*F*(1,22) = 0.35; *p* = 0.56). The frequency of problems reported by patients was significantly lower without a CI compared to the condition with a CI (Fig. 4d).

## Discussion

An interesting aspect of our study was that the SSD-PD patients differed significantly from those tested in previous studies on hearing preservation after a CI. Our patients had successful hearing preservation surgery in one ear and normal (or close to normal) hearing (NH) in the other ear. Despite low frequency residual hearing in their implanted ear, they met the audiological criteria for SSD. In general, patients with SSD can differ significantly in terms of etiology from the usual group of patients with partial deafness (high frequency profound hearing loss in both ears) when considered as candidates for a CI and electric-acoustic stimulation (EAS). For post-lingual SSD, the results of a study done by Usami et al. [22] indicated that idiopathic sudden sensorineural hearing loss (54.6%) was the most common etiology, followed by various forms of otitis media. In contrast, among postlingual PD patients, the most frequent etiology has been found to be genetic (35.8%), followed by otosclerosis (2%), otitis media (2%), and acoustic neuroma (1%) [23]. The possible differences between the etiology of SSD patients and patients with PD motivated us to compare the HP groups, since our SSD patients possessed low frequency residual hearing in the ear scheduled for implantation.

The outcome measure was hearing preservation as determined by the Skarzynski Classification System. The results showed excellent hearing preservation in the SSD-PD group. In this group, mean hearing preservation (HP%) for the implanted ear was 82% one month after CI surgery and 75% 14 months after implantation; in comparison, results in the PD group were 71% and 69% respectively. However, the differences between the groups were not statistically significant. This tends to indicate that any differences in etiology between the SSD-PD group and the PD group were not relevant for hearing preservation after a CI. At the same time, the figures above for hearing preservation in our SSD-PD group were very similar to the results of Snel’s meta-analysis of hearing preservation in a PD group (which, in terms of HP%, were 82.2% after 1 month and 69% after 1 year post-implantation) [3]. Any slight difference could be accounted for by different surgical techniques or types of electrodes rather than difference in etiology between SSD-PD and PD groups.

When assessing the benefit from a CI in cases of SSD, SRT in noise is usually measured for three spatially different locations of speech and noise. This will give one of three possible results: (1) the same SNR in the implanted ear (IE) and the non-implanted ear (NE); (2) more favorable SNR at the IE (IE > NE); or (3) less favorable SNR at the IE (IE < NE) [24]. However, when assessing the benefit of a CI for a PD patient, the most commonly used outcome measures are speech discrimination in quiet and speech discrimination in noise, but only for the condition IE = NE at 65 dB speech presentation level and 10 dB SNR. Therefore, to directly compare the CI benefit identified in our SSD-PD group with that in our PD group (low frequency hearing in both ears), we thought it best to use the APHAB questionnaire, which could assess hearing-related quality of life in both the SSD and PD groups [24, 25]. Since no single measure of audiological outcome predicts the self-reported benefit of a CI, our thinking was that APHAB could provide a single measure of CI benefit appropriate for both groups [26].

The results of the current study indicate that, in terms of APHAB global score, there is no difference in benefit from CI between the SSD-PD and PD groups, a result that also held for the ease of communication and reverberation subscales. However, for the background noise subscale, the benefit for the SSD-PD group was larger than for the PD group. This is understandable by recognising that the ability to discriminate speech in noise depends largely on binaural cues, and in the SSD-PD group a CI restores binaural hearing to a larger extent than in the PD group. In particular, in the SSD-PD group there is a better access to the ILD cue, which is maintained by high frequency hearing through the CI in the implanted ear and natural hearing in the contralateral ear. In the PD group, after unilateral cochlear implantation, access to ILD is limited because of profound high frequency hearing loss in the ear contralateral to the CI.

So far as we know, the benefit of a CI in a SSD-PD group has not been tested, and so our results can only be compared with published benefits of a CI in separate SSD and PD groups. The mean CI benefits in our SSD-PD group—revealed by APHAB global score as well as by ease of communication, background noise, and reverberation subscales—are in general larger than those reported in SSD groups [27–31] or in PD groups [32, 33]. The larger hearing benefit observed in our SSD-PD group might stem from restoration of superior binaural hearing, with the availability of both interaural time difference (ITD) and interaural level difference (ILD) cues. Our SSD-PD patients were unique in that they had successful hearing preservation surgery in one ear and normal or close to normal hearing (NH) in the other. It has already been documented that in such a group, due to hearing preservation in the implanted ear, bilateral acoustic LF hearing provides extra hearing benefits via squelch and redundancy effects (over and above the “better ear” effect) [34]. On the other hand, in the SSD group, binaural benefit is limited due to poor temporal fine structure provided by a CI. In patients with PD in both ears, unilateral cochlear implantation with hearing preservation can provide access to ITD fine structure cues at low frequencies; however, lack of HF audibility in the ear contralateral to the CI removes access to ILD cues [34–39]. Therefore, for the SSD-PD group, access to both types of cues (ITD and ILD) can improve speech performance, especially in noise, leading to increased hearing benefit compared to the PD group, as indicated by the APHAB results.

## Summary

Results of the current study have demonstrated hearing preservation in a unique population of patients who were qualified as having SSD but still had residual hearing in the implanted ear. Hearing benefit was measured to be larger than that in patients with partial deafness in both ears who also underwent unilateral cochlear implantation. We conclude that for a patient with single-sided deafness, residual LF hearing in the ear to be implanted should not be considered a contraindication for receiving a CI.

## Data Availability

The authors have full control of all primary data and that they agree to allow the journal to review their data upon request.
